# Case Report: Holistic approach to management of an infant with severe osteogenesis imperfecta in the neonatal intensive care unit

**DOI:** 10.3389/fped.2025.1475545

**Published:** 2025-06-11

**Authors:** Dipsal Timila, Amna Mousa, Lalitha Gundamraj

**Affiliations:** ^1^ Department of Pediatrics, Pediatric Residency Program, Michigan State University/University of Michigan Health- Sparrow Hospital, Lansing, MI, United States; ^2^ Department of Pediatrics and Human Development, College of Human Medicine, Division of Neonatology, Michigan State University, East Lansing, MI, United States

**Keywords:** severe osteogenesis imperfecta, holistic approach, NICU, newborn, osteogenesis imperfecta type II, lethal skeletal dysplasia, concurrent hospice care, life-limiting dysplasia

## Abstract

**Background:**

Osteogenesis Imperfecta (OI) Type II is the most severe and often lethal form of OI, characterized by profound skeletal fragility and perinatal complications, most notably respiratory failure secondary to thoracic deformities and pulmonary hypoplasia. Effective NICU management requires a holistic, family-centered approach combined with comprehensive medical care, and can be particularly challenging in centers with limited experience managing similar conditions. This is further complicated by ethical dilemmas, including decisions regarding the extent of interventions requiring nuanced judgment and continual reassessment of goals of care.

**Case presentation:**

We describe a term infant prenatally diagnosed with OI Type II, born to an 18-year-old mother. After prenatal counseling from maternal fetal medicine (MFM) and neonatologist about the poor prognosis, the mother opted to continue the pregnancy. At birth, the baby presented with multiple fractures, severe growth restriction and required noninvasive positive pressure ventilation. Management required balancing medical interventions with family goals, the infant's quality of life, and ethical dilemmas due to the life-limiting prognosis. A holistic approach involved early multidisciplinary collaboration, weekly communication of family goals, and consistent medical updates along with supporting the teen mother and her family through complex decisions. This led to a safe discharge to concurrent hospice care with supplemental oxygen via nasal cannula and a feeding tube, alongside comprehensive specialty follow-up.

**Conclusion:**

This case helps expand the scope of what is possible for families facing life-limiting diagnosis. It informs best practices for navigating prognostic uncertainty, guiding ethical decision-making, and promoting holistic support beyond survival metrics. Cases like this advocate for a shift in focus from solely survival to quality of life and help establish thoughtful standards for managing severe skeletal dysplasias within perinatal and neonatal care frameworks.

## 1 Introduction

Osteogenesis Imperfecta (OI) is a heterogeneous group of connective tissue disorders caused by mutations in collagen-related genes, characterized by skeletal abnormalities leading to bone fragility and deformity (1). Severity of OI varies widely, from mild forms to severe, perinatally lethal conditions, with a prevalence of approximately one in 15,000 to 20,000 births (1). A recent classification system includes rarer forms (Table 1). OI type II is a lethal form of OI. Patients with mild OI experience minimal impairment and maintain high functionality, while those with severe forms face significant morbidity and mortality, due to pulmonary complications (1). Survivors face mobility impairments that may require orthopedic surgery. Bisphosphonates increase bone volume and strength, reducing fracture risk (2).

**Table 1:** A detailed overview of the genetic and clinical heterogeneity of osteogenesis imperfecta (OI), categorizing the condition based on defects in collagen structure, folding, bone mineralization, and other related pathways. This classification aids in understanding the broad spectrum of OI, from mild to severe forms, and highlights the genetic diversity underlying the disorder. Adapted with permission from Osteogenesis Imperfecta by Forlino and Marini (1). Licensed under (source Elsevier - Lancet 2016).

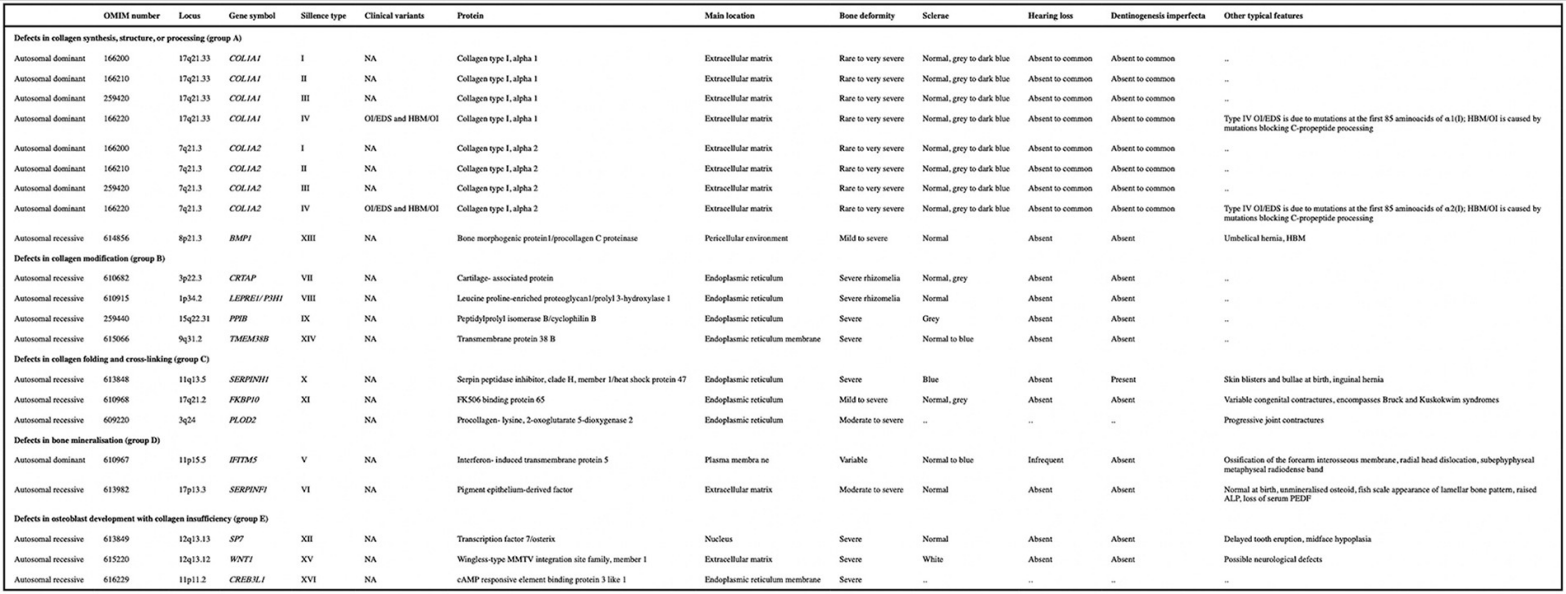

OMIM, online mendelian inheritance in man; NA, not applicable; OI, osteogenesis imperfecta; EDS, Ehlers-Danlos syndrome; HBM, high bone mass; ALP, alkaline phosphatase; PEDF, pigment epithelium-derived factor; MMTV, mouse mammary tumour virus.

Advances in prenatal and postnatal diagnosis, neonatal care, and genetic testing have led to improved survival rates (3). Comprehensive guidelines for managing newborns with severe OI in NICUs are scarce. Prognostication of survival during the perinatal period is challenging, raising ethical questions about the extent of medical interventions. This case report contributes to the limited literature on prenatally diagnosed severe OI highlighting medical, ethical, and psychosocial complexities, and the support required for a seamless transition to concurrent hospice care.

## 2 Case description

A term infant born to an 18-year-old primigravida whose 18-week fetal anatomy scan revealed micromelia and severely shortened long bones. The femoral length was 16.3 mm and femur to abdominal circumference ratio was 0.11 suggesting severe skeletal dysplasia. She was counselled regarding extreme bone fragility, *in utero* fractures and poor prognosis, and elected continuing pregnancy. Prenatal cell free DNA testing (Vistara) identified a COL1A2 gene mutation, consistent with Osteogenesis Imperfecta (OI) Type II. The remainder of her prenatal course was unremarkable except for mild anemia due to sickle cell trait. Family history included Huntington's disease and asthma, and no known history of skeletal dysplasia.

A 35-week ultrasound revealed multiple fractures involving the ribs and long bones. The differential diagnoses included thanatophoric dysplasia, achondrogenesis, hypophosphatasia, and OI Type II. Prenatal counselling by the neonatologist included the high likelihood of neonatal death, poor long-term outcomes, respiratory complications, and risk of rib fractures from chest compressions and cardiopulmonary resuscitation, if necessary.

Mother declined chest compressions. She was primarily supported by her parents and expressed a preference to receive all care at the delivery hospital. She delivered via elective C-section at 37 weeks to minimize fracture risk. At birth, the infant was vigorous with severe growth-restriction and features consistent with OI. She received delayed cord clamping for 30 s, required positive pressure ventilation for respiratory distress, and admitted to the NICU. Apgar scores were 6 and 9 at 1 and 5 min, respectively. Vitals were stable. Birth anthropometrics included a weight of 2.015 kg (Z = −3.05), Length of 34 cm (Z = −8.33), and head circumference of 32 cm (Z = −2.18). Initial respiratory support included nasal intermittent positive pressure ventilation (NIPPV) with peak inspiratory pressure (PIP) of 30 cm of H2O, positive end-expiratory pressure (PEEP) of 5 cm H2O, and an FiO2 of 1.0.

**Figure 1:**
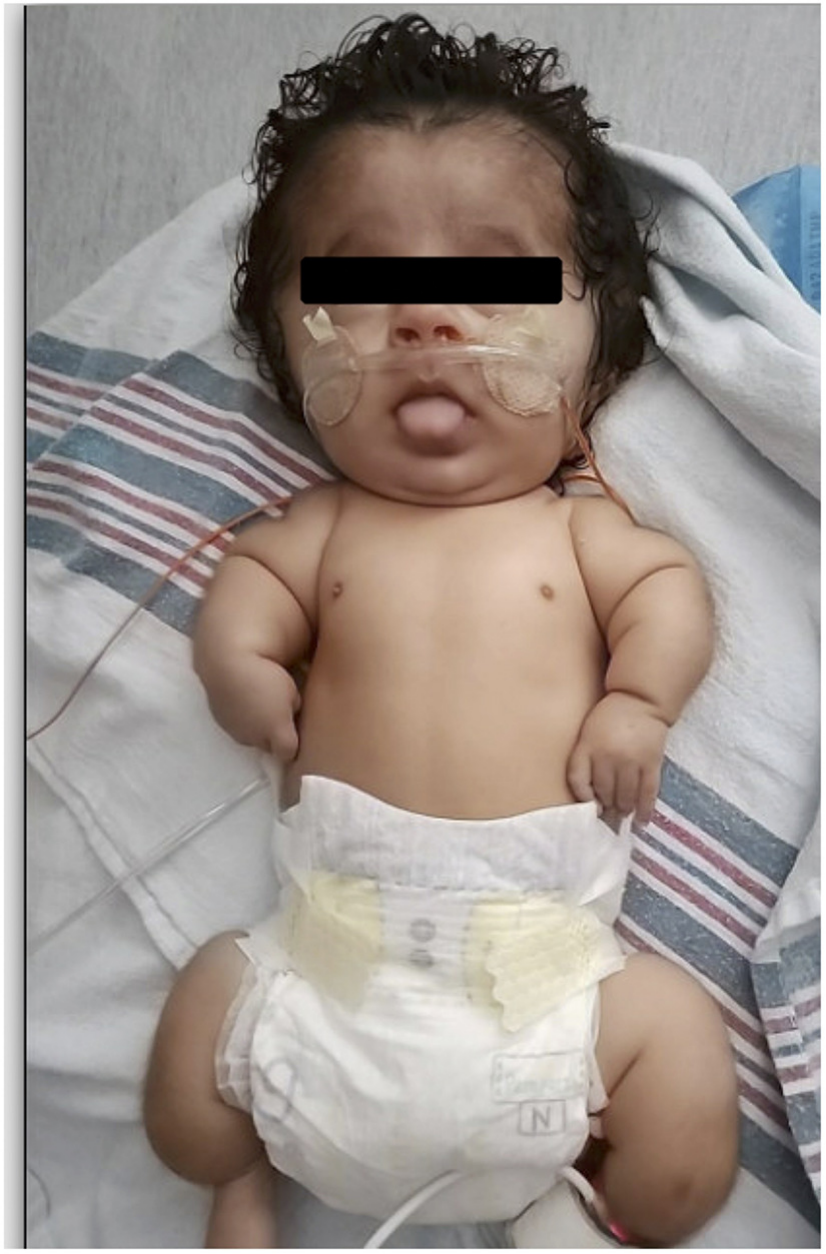
Photograph of infant at 3 months of age (published with consent from mother of baby).

Physical exam revealed soft cranial bones, widely splayed cranial sutures, a broad forehead, blue sclera, low set ears, micrognathia, midface hypoplasia, bilateral single palmar creases, a shortened trunk, and short and bowed long bones. Spontaneous limb movement was observed (see Figure 1).

**Figure 2:**
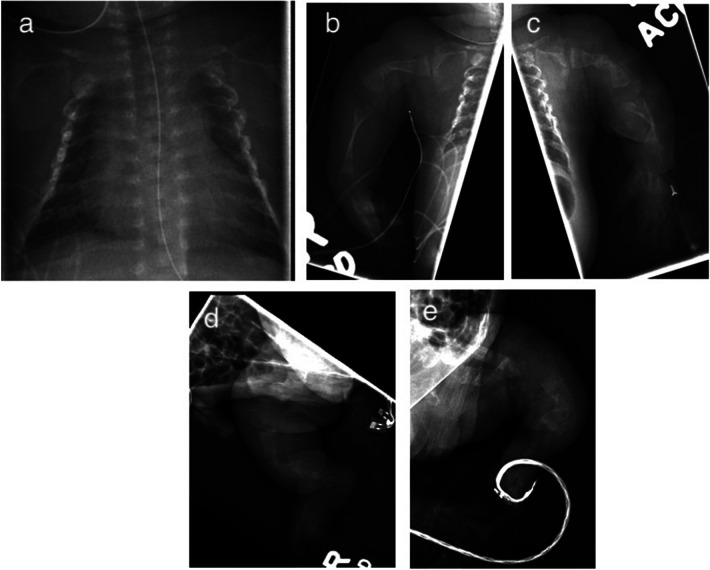
Initial x-ray demonstrating multiple bilateral rib fractures **(a)** and long bones **(b–e)** with curvilinear configuration and irregularities consistent with various stages of healing.

Skeletal survey revealed a bell-shaped chest, multiple fractures at various stages of healing, along the lateral aspects of several bilateral ribs, and irregularities of the bilateral curvilinear femurs, humeri, radii/ulnae, and tibiae/fibulae (Figure 2). Serum calcium and phosphorous levels were within normal limits. Alkaline phosphatase (ALP) was low at 156 U/L (reference range 169–372 U/L). Echocardiogram and cranial ultrasonography were normal. Targeted genetic testing confirmed a monoallelic pathogenic variant in COL1A2 gene: *c.1783G* *>* *T (p.G595C)*. The mutation was not identified in the maternal blood sample. The presence of this pathogenic variant, and the infant's clinical presentation, confirmed the diagnosis of OI Type II.

The patient was weaned from NIPPV to CPAP on day of life (DOL) 18, transitioned to nasal cannula on DOL 25, and remained on it until discharge. The care team included the NICU team, physical therapist (PT), occupational therapist (OT), bedside nurse, social worker, physical medicine and rehabilitation specialist, hospice physician, hospice nurse, geneticist, pediatric orthopedics and pulmonologist.

Although the infant was clinically stable, an umbilical venous catheter (UVC) was placed to provide total parenteral nutrition (TPN), medications and minimize manipulation and fracture risk during attempts to establish intravenous access. On DOL 2, expressed breast milk feeds were initiated via nasogastric (NG) tube. Total fluid intake was restricted to a maximum of 110–130 ml/kg/day with a target weight gain of 5–10 grams per day to avoid excess adiposity. She was started on calcium, phosphorus, and vitamin D supplementation, with serum levels monitored and dosages adjusted to ensure adequacy. The UVC was removed on DOL 3, once NG feeding was established, facilitating early maternal bonding. By the end of the first week of life, with nursing support, the mother became confident in managing the infant's care needs.

Pain was monitored using the Neonatal Pain, Agitation and Sedation Scale (N-PASS). Adequate pain control was achieved through intravenous and oral morphine (0.05–0.1 mg/kg/dose every 4 h as needed), then weaned to oral acetaminophen (15 mg/kg/dose QID). Gabapentin was started at a dose of 7.5 mg/kg/dose three times a day and gradually increased to a maximum dose of 60 mg/kg/day divided every 6 h for optimal pain management. No side effects were observed. Liver function test closely monitored was normal.

Intravenous pamidronate infusions were administered over four hours beginning on DOL 13, starting at 0.25 mg/kg on the first day, followed by 0.5 mg/kg on DOL 14 and 15. Infant tolerated these well with neither increase in pain medication nor episodes of respiratory distress, fever, or flu-like symptoms. Post-infusion hypocalcemia was observed and managed with oral calcium supplementation.

The infant failed the hearing screening test bilaterally and was referred to audiology for further evaluation and to plan necessary interventions. She remained hemodynamically stable, passed the car bed test, newborn metabolic screening results were normal, and received the Hepatitis B vaccine. Her primary care physician saw her during the week before discharge to establish continuity of care.

On DOL 41, she was discharged to concurrent hospice care with supplemental oxygen via nasal cannula at 2 L/min with FiO2 1.0, and with an NG tube in place for feeding. She was receiving breast milk fortified with Similac Neosure to 22 kcal/oz, with a total fluid goal of 130 ml/kg/day. The mother was trained in the use of all feeding and respiratory support equipment/supplies and demonstrated comfort and competence in managing her infant's care at home. She was instructed on using gel positioning devices and safe holding techniques to minimize the risk of new fractures. Specific swings were trialed and demonstrated to the family for safe use at home.

Post-discharge, the patient is receiving home hospice care. Now 12 months old, she remains on NG tube feeds, weighs only 3.24 kg, with poor weight gain attributed to her chronic condition. She is on home oxygen at 0.5–1 L/min. Developmentally, she can reach for toys and enjoys watching cartoons. Her hospice care includes regular visits from a hospice nurse, medical social worker, and a pastor. A physician with dual training in physical medicine and rehabilitation and palliative care is closely involved in her care.

She received four cycles of pamidronate at a dose of 0.5 mg/kg/day, administered over three consecutive days every three months, with no new fractures since birth. She was admitted and discharged from the pediatric ward and PICU multiple times due to desaturation episodes associated with viral upper respiratory infections. Her pain is well-controlled with acetaminophen, gabapentin, and as-needed morphine. At 8 months of age, she was diagnosed with hydrocephalus, for which a palliative ventriculo-peritoneal (VP) shunt was placed.

## 3 Diagnostic assessment

All diagnostic assessments and therapeutics interventions are discussed in detail in case description above.

## 4 Discussion

Most skeletal dysplasias occur without known risk factors, and some cannot be accurately diagnosed in-utero. Osteogenesis imperfecta (OI) accounts for approximately14% of life- limiting skeletal dysplasias (4). Fetuses with perinatal lethal OI type II are typically identified between 18 and 20 weeks of gestation, characterized by short, crumpled long bones and marked hypomineralization of the facial and skull bones. Approximately 80% of infants with OI type II die within the first week of life (5). The mutation detected in our patient *c1783G* *>* *T (pGly595Cys)* which is a missense variant in the COL1A2 gene has been reported in literature (6). The mutation was not found in the control population. Predictors of lethality include bell shaped thorax, short ribs, severe femoral shortening (>4 standard deviation), a femur length to abdominal circumference ratio < 0.16, a chest to abdomen ratio < 0.6, bone bowing and multiple bone fractures (4).

With early diagnosis and advances in neonatal care, more babies with OI Type II survive the newborn period and beyond. Management remains challenging, underscoring the importance of early, clear communication with families about the severity of the condition and expected outcomes. Providers must understand family goals through collaborative consultations involving maternal fetal medicine (MFM), neonatology, and pediatric palliative care (PPC) to support families throughout their child's journey. Families should be counselled on the potential variability in disease severity, which may evolve with clinical progress, allowing for shared decision-making regarding expectant management. Early evaluation of the family's support system and the availability of local and state resources is essential. Infants with complex life-limiting conditions often require ongoing support, including oxygen, feeding or gastrostomy tubes, respiratory equipment, and monitoring devices. Caregivers must be equipped to manage all aspects of their child's care.

Shared decision-making should begin prenatally and address the potential benefits and harms of interventions, including consideration of “AND” (Allow Natural Death) orders due to the high risk of fractures from chest compressions. Regularly scheduled care conferences with parents should be organized based on medical needs and the family's need to understand their child's progress and care requirements.

### 4.1 Pain management

Pain from fractures can significantly impact a newborn's respiratory status. Several neonatal pain assessment scales exist, each with varying validation and clinical applicability. Selection should be guided by validation in the local language, clinical context, and the healthcare team's familiarity with the tool. The NPASS (Neonatal Pain, Agitation, and Sedation Scale) is validated for term and preterm neonates and is practical for monitoring pain, agitation, and sedation in NICU settings (7).

Opioids may be administered for diagnostic assessments and procedures, based on the severity of fractures and NPASS score. Dosing should be optimized to prevent respiratory depression and side effects such as constipation, while minimizing respiratory splinting to maintain respiratory efficiency. The goal should be to wean opioids once clinical stability is achieved (2).

Non-opioids like acetaminophen and gabapentin are effective alternatives due to lower risk of side effects. Liver function should be monitored. Gabapentin is typically dosed at 2.5–10 mg/kg/day, up to 35 mg/kg/day. In our case, a higher dose of 60 mg/kg/day was used after consultation with the palliative care physician and review of literature confirming safety (8). This helped wean the patient off morphine and achieve adequate pain control without significant adverse effects.

Delicate handling is essential to prevent new fractures. Egg crate or gel mattresses are recommended. Hands-on care, such as repositioning and diaper changes, should be clustered to limit physical manipulation.

### 4.2 Respiratory support

Fear of cervical fracture should not preclude necessary respiratory support in patients with severe OI, provided appropriate precautions are taken (2). The care team should be prepared for endotracheal intubation or video laryngoscopy, ideally performed by the most experienced team member in the delivery room or NICU to maximize success. A laryngeal mask airway (LMA) may serve as a suitable alternative to invasive intubation.

Understanding local capabilities for respiratory support is essential to ensure a safe transition to concurrent hospice care, given the risk of pulmonary complications. This includes arranging specialized equipment such as home ventilators, CPAP machines, or nasal cannulas, and training caregivers on proper equipment use and recognizing signs of respiratory distress.

### 4.3 Nutritional support

A lower target for weight gain-approximately 5–10 gm/day has been recommended for patients with OI, achieved by limiting total fluid intake to 100–110 ml/kg/day and providing 75–80 kcal/kg/day. This helps prevent excess adiposity, which may result from decreased physical activity and lower caloric needs in these patients (2). Excessive fat deposition, abdominal competition, and fluid retention can contribute to respiratory compromise. The nutritionist should monitor growth to avoid abrupt changes that may affect respiratory status. Early supplementation of calcium, phosphorus, and vitamin D with enteral feedings supports optimized growth and bone strength.

### 4.4 Bone density management

Pamidronate inhibits bone resorption, improving bone density, stability, and reducing bone pain in children with moderate to severe OI (9). Various dosing regimens and frequencies have been described (10). A three-day consecutive dosing schedule using varying doses has been reported, with the first dose reduced to half to minimize the risk of acute febrile phase reaction and respiratory distress. We administered a lower dosage of 0.125 mg/kg/day on the first day, followed by 0.25 mg/kg/day on days two and three, to reduce side effects in the initial treatment cycles (11). Higher dose regimens, such as 0.5 mg/kg/day on day one followed by 1 mg/kg/day on days two and three, are described. Monthly, bimonthly, and quarterly pamidronate cycles have been used based on clinical need and tolerance (9, 11).

### 4.5 Genetic counseling

Initial diagnosis of a lethal form of OI requires regular fetal reevaluation throughout pregnancy and just before delivery. Studies have shown that the specific type of OI for an individual should not be determined solely by prenatal findings or genetic testing, but rather by the clinical course over time, as severity may vary. For example, a patient with a previously reported lethal COL1A1 mutation had a non-lethal course and survived beyond 22 months of age (12). Therefore, prenatal counseling should include discussions about expectant management strategies and the potential variability in disease severity and long-term developmental outcomes. Initial genetic counseling should address inheritance patterns, recurrence risk, and family planning, with follow-up after discharge to monitor progress and adjust the care plan as needed.

### 4.6 Hospice and palliative care support

Concurrent care is now a federally mandated option for children and their families in the USA. Section 232 of the 2010 Patient Protection and Affordable Care Act (ACA), titled *Concurrent Care for Children*, allows children enrolled in Medicaid or the Children's Health Insurance Program (CHIP) in all U.S. states to receive hospice services while continuing curative therapies (13). Many pediatricians, specialists, hospice clinicians, and families may be unaware of this option. Although pediatric palliative care (PPC) and hospice care are often used interchangeably, PPC is a specialized service, focusing on symptom relief, emotional and psychological support to families, and end-of-life planning when necessary for children living with serious, life-limiting illnesses.

### 4.7 Ethical considerations

Parents experience ethical dilemmas, anguish, and stress when faced with diagnosis of life-limiting conditions. They must make life-altering decisions based on limited information, often oscillating between pursuing all possible interventions and limiting aggressive treatment. Involvement of a multidisciplinary team from the prenatal period is essential to understand parental preferences and identify necessary support.

A teenage mother faced with a prenatal diagnosis of severe skeletal dysplasia was informed of the poor prognosis but wished to continue the pregnancy and pursue resuscitation (excluding chest compressions). Intubation was a concern due to the risk of cervical injuries from neck manipulation. The hospital ethics committee was consulted, and a literature review revealed no reports of cervical fractures in similar patients (2). The team proceeded with an AND (Allow Natural Death) order excluding chest compressions. Neither chest compressions nor intubation were required during the infant's hospital stay.

Providers struggle to balance beneficence (acting in the patient's best interest) with non-maleficence (avoiding harm) in complex cases where the benefit of intervention is uncertain (14). They may experience moral distress if they believe certain interventions could prolong suffering. Group consensus and open communication are essential, emphasizing evolving clarifications and input from junior team members who may feel hesitant to voice concerns.

Regular care conferences with the mother, her family, the NICU team, and the multidisciplinary team were crucial for safe transition. Healthcare providers must engage in respectful communication and collaborate with families while upholding autonomy, beneficence, and non-maleficence. Chaplains can offer comfort, especially when religious beliefs influence decisions regarding interventions and end-of-life care (15).

## 5 Parent perspective

Mother later reported experiencing fear when first informed about her baby's life-limiting illness, and was anxious before delivery, as she did not expect the baby to survive, but felt relief upon hearing the baby cry. Her primary goal was to take her baby home, regardless of condition. Discussions around code status caused distress, although she appreciated the information. Given her level of comprehension and willingness to be involved, she was encouraged to provide early skin-to-skin care and participate in all aspects of care to help her cope with stress and feel empowered as an important part of her child's health.

As an 18-year-old, the mother relied heavily on her parents and siblings for support. We ensured their presence during all weekly discussions, especially before discharge. Despite her young age, she was fully capable of making informed decisions for her child, with family support always available when needed.

## 6 Conclusion

Our patient's case challenges assumptions about uniform lethality and underscores the value of shared decision-making, symptom-guided medical management, and a flexible, family-centered approach. Early multidisciplinary involvement of maternal-fetal medicine, neonatology, pediatric palliative care, ethics, and hospice allowed for balancing medical realities and the mother's goals.

## Data Availability

The original contributions presented in the study are included in the article/Supplementary Material, further inquiries can be directed to the corresponding author.
